# Barriers to and facilitators of healthcare professionals in ADR reporting in a tertiary care hospital in India

**DOI:** 10.1186/s12913-024-12139-w

**Published:** 2025-01-28

**Authors:** Ian Osoro N, Suhaib KP, Jamuna Rani R, Rajanandh MG

**Affiliations:** 1 Department of Pharmacy Practice, SRM College of Pharmacy, SRM Institute of Science and Technology, Kattankulathur , Tamil Nadu, 603203 India; 2 Department of Pharmacology, SRM Medical College Hospital and Research Centre, SRM Institute of Science and Technology, Kattankulathur , Tamil Nadu, 603203 India

**Keywords:** Adverse drug reaction, Pharmacovigilance, Clinical pharmacy, Physicians, Nurses

## Abstract

**Introduction:**

Several adverse drug reactions (ADRs) go unreported within a healthcare setting despite the risks they cause. We therefore decided to conduct this study in order to recognize the obstacles that hinder the healthcare professionals (HCPs) in a tertiary care hospital in Kattankulathur, Tamil Nadu from reporting ADRs and what strategies ought to be implemented.

**Methods:**

We carried out a cross-sectional study among the HCPs such as doctors, pharmacists and nurses within our institution. A pre-validated questionnaire was used to collect data on the socio-demographics, barriers and facilitators in reporting ADR. A 2 weeks timeline was given to the HCPs to fill the questionnaire forms. Out of the 107 forms distributed, we received 80 of them that were duly filled. Data was analyzed using IBM SPSS version 26.

**Results:**

Out of the 80 HCPs, only 22 of them had reported any ADRs in their career. 52% of our HCPs reported the lack of understanding of ADR reporting mechanism as their main hindrance. Additionally, 25 (31%) of the HCPs stated that reporting ADRs is time consuming. 18 (22%) of them reported a fear of legal liability. 13 (16%) of them stated that the reporting from is complicated and 29 (36%) stated a lack of motivation as the reason for not reporting ADR. Majority of our HCPs 76 (95%) recommended the need for continuous medical education and training as the best strategy to improve ADR reporting.

**Conclusion:**

Barriers such as time constraints, workload pressures and competing priorities often hinder HCPs from dedicating adequate attention to ADR reporting. The inclusion of topics related to ADR reporting in the curriculum (i.e. clinical pharmacology) and increased awareness from the ADR monitoring centre were seen to be significant facilitators to enhance ADR reporting among health care practitioners.

**Supplementary Information:**

The online version contains supplementary material available at 10.1186/s12913-024-12139-w.

## Introduction

Pharmacovigilance is the process of ensuring the safety of medications by taking the necessary actions to minimize risks and enhance efficacy of the drugs. The Pharmacovigilance Programme of India (PvPI) currently runs all pharmacovigilance related activities in India. Some of these activities include: assessing the risk–benefit ratio of currently marketed medications, identify and report new signals as seen in various reporting centres and enhance the use of rational medication use among others. The PvPI started in 2010 and it’s under the ambit of the Ministry of Healthcare and Family Welfare (MOHFW). Prior to the introduction of the PvPI, a well-structured system to monitor ADRs was already in place since 1986 [1]. ADRs are harmful medication effects although they are usually unintentional. These reactions may have fatal outcomes hence the need to report any ADRs noticed in patients. Through the history of medical evolution, certain ADRs have happened that have led to the development of better ways to review medications. An example is the thalidomide tragedy that happened in the 1960s and led to the development of phocomelia and this medication was subsequently withdrawn from the market as a morning sickness drug thereby preventing more children from having similar consequences [2]. The benefit of ADR reporting can be seen in identifying rare cases particularly in the post marketing surveillance (PMS) period since majority of new medications are approved if they can show a higher benefit compared to the risk it poses in treating a condition [3].

Identification of ADRs that arise soon after taking a medication is easier compared to those related to long term use of a medication. Recurrence is therefore one of the ways to validate whether a symptom observed is as a result of a particular drug consumed although this is restricted in cases of serious adverse events. MedWatch is a safety information and online ADR reporting program that can be used by anyone. Medical professionals are encouraged to use the US Food and Drug Administration (USFDA) Adverse Event Reporting System (FAERS) which is a search engine that gives information on ADRs [4].

Globally, ADRs are suspected to be the fourth to sixth leading cause of death and it mainly affects individuals in low-income countries and vulnerable populations. Additionally, ADRs have been noted to have a great financial burden and increased healthcare costs. In US, it is estimated that around $30 billion is used annually to tackle ADR related conditions. A study conducted in an Irish tertiary care centre revealed that hospital related ADR admission costs were €9538 and an increase was seen in more severe cases [5, 6].

In 1968, the Programme for International Drug Monitoring was initiated by the WHO and currently, the number of member countries have increased to 180 (159 full members and 21 are associates). The aim of the programme is to present all serious ADRs particularly of medications already in the public in a systematic way using the VigiBase (a WHO database for medicinal adverse events) [7]. A study by Gidey et al. conducted a study in Ethiopia showed that the level of ADR reporting was low among their HCPs with lack of knowledge, little experience and lack of training noted as major barriers. The authors concluded that enhancing trainings particularly among the inexperience HCPs will have a significant impact on their ADR reporting practises [8].

Results from a systematic review showed that attitudes such as ignorance, insecurity and lethargy were associated with low ADR reporting and a study by Schory et al. showed that equipping HCPs with relevant ADR reporting information will improve ADR reporting. Having a pharmacovigilance specialist will additionally be helpful as they will use reminders to encourage other HCPs to report ADRs [9, 10]. Lack of knowledge has been found to be a key stumbling block in ADR reporting however this can be overcome by improved understanding of ADR through creating awareness and increased knowledge. HCPs have noted the lack of time as an issue contributing towards lack of ADR reporting and thereby having specialist who will help reporting the ADRs will be great.

Additionally, having a better functioning ADR online reporting system will increase the likelihood of more ADR reports from HCPS as less time will be consumed in the registration and reporting. Low motivation is also an integral barrier in the reporting of ADR since most HCPs feel they are not recognised for their efforts hence the need for medical facilities to encourage their HCPs through different means i.e. incentives which have been seen to raise the ADR reports [11–13].

In India, the National coordinating centre of PvPI in Ghaziabad has come up with various ways through which ADR can be reported by HCPS, patients, consumers or a layman. This include: ADR reporting forms available in ADR Monitoring Centres (AMCs) or can be downloaded through the Indian Pharmacopoeia Commission (IPC) or Central Drugs Standard Control Organisation (CDSCO) websites, ADR-PvPI mobile application and calling toll-free number, i.e., 1800–180–3024. The forms are designed to be easily usable by anyone and the voluntary reporting by Non-Adverse Drug Reaction Monitoring Centres through email (icsr.nccpvpi@gmail.com) is highly encouraged. Further, the reporting forms have been translated to 10 regional languages such as Marathi, Hindi, Telugu, Gujarati, Oriya, Tamil, Malayalam, Bengali, Kannada and Assamese [14]. ADRs might have a minor, intermediate, serious or life-threatening outcomes if not carefully handled. Some of the currently available methods in treating ADRs include: dose adjustment, treatment/medication cessation and switching to alternative medication [15, 16]. A few factors have been noted to increase the chances of experiencing an ADRs i.e.: age (geriatric or young), concurrent use of various drugs, hereditary factors or lactation [17–19].

Underreporting of ADRs has been noted particularly in rural India due to factors such as lack of proper reporting systems. A study conducted by Tandon et al. showed that among 90 AMCs, 68% and 81% of physicians and pharmacists respectively were not aware of the PvPI. Moreover, fewer than 1% ADR cases are reported in India in comparison to 5% at the global scenario hence the need to assess the facilitators and barriers which may influence the HCPs practices and attitudes [20]. Gupta et al. assessed factors responsible for ADR underreporting among resident doctors and 80% of the participants lacked understanding of the ADR reporting system hence the low level of awareness. Also, lack of time was seen to be a major contributor for not reporting ADRs (70%). The authors concluded that using effective systems along with having an active National programme would enhance ADR reporting [21].

Another study by Thakare et al. conducted a five-year analysis of ADR reporting in their tertiary care hospital in Navi Mumbai. It was found out that the number of female ADR cases were more than the male i.e. 58.6% and 41.4% respectively. Furthermore, they noted that antibiotics contributed to the most cases reported (22.11%). Importantly, conducted sensitization programmes regrading ADR reporting was seen to bring a positive pattern in the cases reported over the five-year period [22]. Sharma et al. analysed the occurrence of spontaneous reporting of ADRs in an AMC in Madhya Pradesh, Central India. This 7-year study showed that 1980 reports were received and antimicrobials were the highest contributor of the ADRs i.e. 29%. Particularly, zidovudine was responsible for 88% of cases reported and majority of the ADRs (28%) affected the skin. The authors concluded that ADR underreporting was found in their study and interventions such as trainings may improve ADR reporting [23]. A study by Seenivasan et al. had similar findings where antibiotics were the most frequent drug category of ADRs reported and rashes were the common ADR noted [24]. Despite the availability of few studies on barriers and facilitators of ADR reporting in India, there is still a greater need for more studies particularly with the difference between rural and urban settings [25].

The ADR monitoring centre in our institution is under the PvPI programs and its actively involved in spreading awareness not only among HCPs but also the surrounding society. In collaboration with the National PvPI, we conduct various events such as pharmacovigilance celebration week among other programs. As a result, an increase has been seen in the number of cases being reported i.e. from 120 cases in 2023 to 207 cases in 2024 (January to September).

### Role of HCPs in ADR monitoring and management

Physicians have a critical role in the implementation of any pharmacovigilance systems. They can be involved in detecting ADRs when conducting clinical trials or during their clinical practice. Further, submission of Periodic Safety Update Reports (PSURs) has been greatly encouraged among doctors [26]. A study by Upadhyaya et al. concluded that a close working relationship between doctors and AMCs will be very beneficial [27]. Pharmacists are well trained individuals on pharmacotherapy hence they have a crucial role in correct identification and reporting of ADRs [28]. Hospital as well as community pharmacists mainly dispense medications to patients and through their interaction with patients during counselling, they are able to detect and report ADRs is important [29]. Nurses are normally the first point of contact with the patients hence they are crucial in the ADR reporting, training and creating awareness. Moreover, through their help, many patients can be taught on detecting and reporting any ADRs based on their medical conditions. However, studies have shown that ADR reporting from nurses has been found to be low [30]. With this background, the present study aimed to identify the barriers and facilitators for ADR reporting among HCPs in a tertiary care hospital in Tamil Nadu, India.

## Materials and methods

### Study setting

The study was conducted among HCPs (doctors, pharmacists and nurses) in SRM Medical College and Hospital, Kattankulathur which is a tertiary healthcare facility in Tamil Nadu state, India.

### Study design and population

We conducted a cross-sectional study among HCPs for a period of six months (October 2023 to March 2024). Inclusion criteria for this study was practising HCP in SRM Medical College Hospital. The HCPs not willing to participate were excluded from the study as per recommendation by the ethics committee. Written informed consent was obtained before the start of data collection. STROBE guidelines were followed in the data collection.

### Sample size and sampling technique

The sample size was calculated by assuming a 95% confidence interval and a 5% level of significance and margin of error as per the literature [31]. The Sample Size Formula = $\lbrack\mathrm z^2\;\ast\;\mathrm p(1-\mathrm p)\rbrack\;/\;\mathrm e^2\;/\;1\:+\:\lbrack\mathrm z^2\;\ast\;\mathrm p(1-\mathrm p)\rbrack\;/\;\mathrm e^2\;\ast\;\mathrm N\rbrack$ was used where, N is the population size, z is the z-score (1.96), e is the margin of error (0.05), and p is the standard of deviation (0.5). We used convenience sampling technique in this study.

### Study variables

i) Dependent variables included: Understanding of HCPs regarding ADR reporting and Barriers and strategies of reporting ADRs
ii) Independent variables included: (a) Age, gender, profession

### Data collection tool development

We developed our questionnaire after going through the literature of various related studies [32–35]. Demographic details and details regarding conversance with barriers and facilitators of ADR reporting in clinical practice were incorporated. The tool was divided into three main sections. The first section included demographic details of participants i.e. age, gender, designation, work experience, profession and information regarding awareness of the AMC within the institution. The second section contained information on the barriers HCPs face when reporting ADRs. The third section included the facilitators that can enhance reporting of ADRs by HCPs (Supplementary file).

### Validation of data collection tool

Two external experts (scientists from the ICMR—National Institute of Epidemiology) verified the questionnaire focusing on its relevance and accuracy and gave suggestions which were then included in the final tool. 15 HCPs were used for pilot testing and were subsequently excluded from study. The internal consistency of tool was 0.78.

### Statistical analysis

Analysis was conducted using IBM SPSS Statistics; Software version: 26. The results were presented as frequencies and percentages or mean ± SD for categorical and continuous variables respectively. Kruskal Wallis test and Mann Whitney U tests were used and a *p-* value less than 0.05 indicated significance.

## Results

Out of the total 107 questionnaires that were circulated among HCPs in SRM Medical College Hospital, only 80 of them were filled and submitted thus we had a response rate of 74.7% in our study. Further, the baseline characteristics of the study population are presented in Table 1.

**Table 1:** Demographic details of study participants

Characteristics	Total N (%)	Doctors n (%)	Pharmacists n (%)	Nurses n (%)
**Gender**				
Male	45 (56.25%)	35 (43.75%)	5 (6.25%)	5 (6.25%)
Female	35 (43.75%	15 (18.75%)	10 (12.5%)	10 (12.5%)
**Age (in years)**				
18–25	34 (42.5%)	13 (16.25%)	9 (11.25%)	12 (15%)
26–35	31 (38.75%)	26 (32.5%)	3 (3.75%)	2 (2.5%)
36–45	9 (11.25%)	5 (6.25%)	3 (3.75%)	1 (1.25%)
Above 45	6 (7.5%)	6 (7.5%)	0	0
**Awareness of AMC in the institution**				
Yes	66 (82.5%)	40 (50%)	13 (16.25%)	13 (16.25%)
No	14 (17.5%)	10 (12.5%)	2 (2.5%)	2 (2.5%)
**Work experience (in years)**				
0–5	42 (52.5%)	30 (37.5%)	6 (7.5%)	6 (7.5%)
6–10	26 (32.5%)	13 (16.25%)	7 (8.75%)	6 (7.5%)
11–15	6 (7.5%)	3 (3.75%)	0	3 (3.75%)
16–20	4 (5%)	2 (2.5%)	2 (2.5%)	0
Above 20	2 (2.5%)	2 (2.5%)	0	0

### Socio-demographic characteristics of participants

The mean age of the respondents was 29.27 years. The majority of the participants came under the age category between 18–25 years that is 34 (42.5%) participants and the minority were in the age category above 45 years that is 6 (7.5%) as shown in Table 1. Moreover, the age categorization of the HCPs, their gender distribution and awareness of ADR centre have been shown in Table 1*.*

### Barriers of ADR reporting

**Fig. 1:**
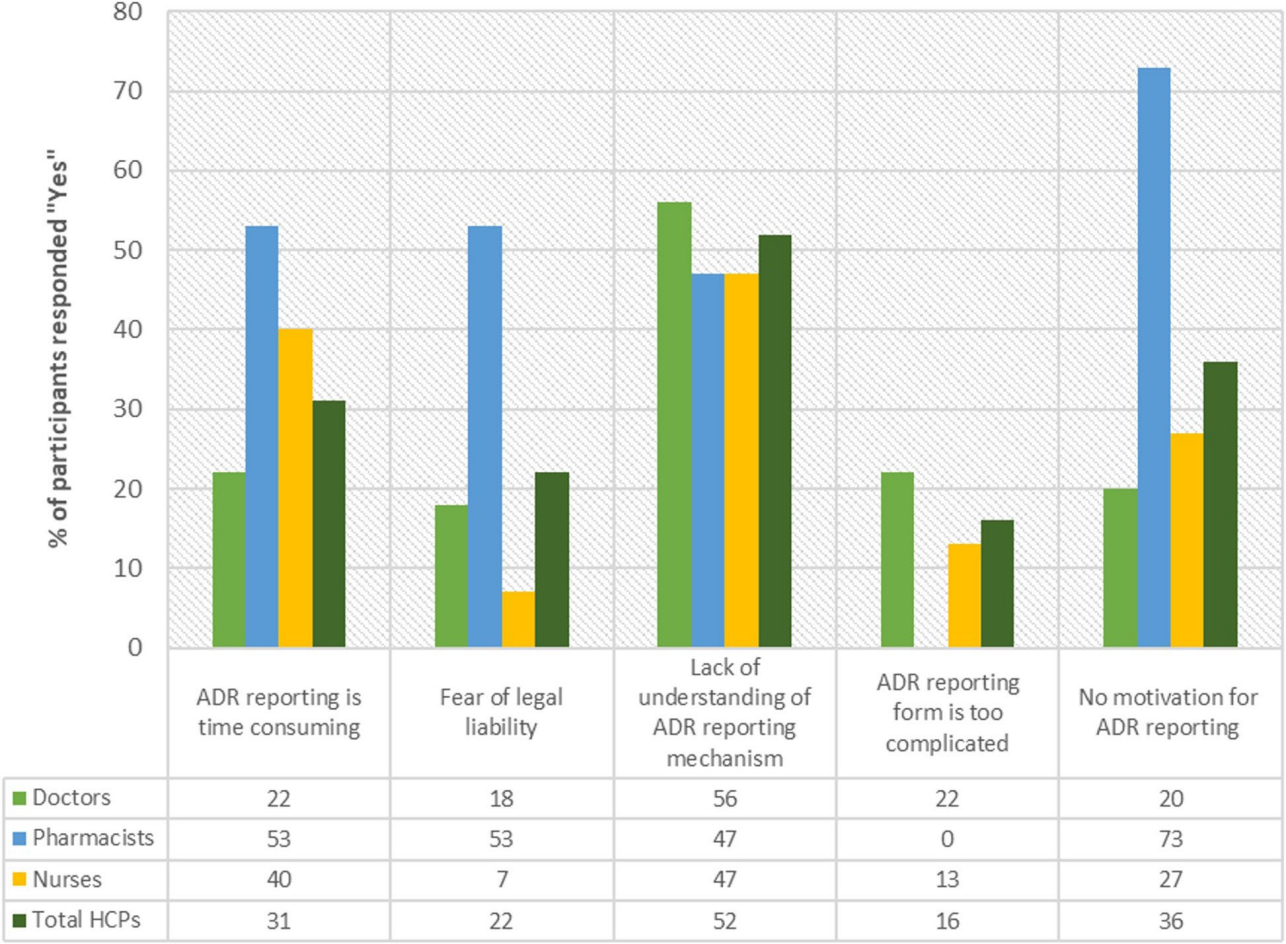
Barriers in reporting ADRs

In collecting data regarding ADR reporting barriers, five barriers were given in the questionnaire as shown in Fig. 1 i.e. “reporting ADR is time consuming”, “fear of legal liability”, there is a “lack of understanding of the reporting mechanisms”, “reporting form is too complicated” and “no motivation”. The prevalence of HCPs who believed reporting ADR is time consuming was 25 (31%) and 55 (69%) did not agree that ADR reporting is time consuming. Out of the 25 who believed ADR reporting was time consuming, 11 (22%) were doctors, 8 (53%) were pharmacists and 6 (40%) were nurses. We discovered that 18 (22%) of the HCPs had a fear of legal liability and it acted as a barrier to ADR reporting. Out of the 15 pharmacists, 8 (53%) stated that the reason for not reporting ADR is fear of legal liability. Additionally, 9 (18%) doctors and 1(7%) nurse stated the same reason.

A total of 42 (52%) HCPs stated that they lacked understanding of the reporting mechanism in this study. Out of the 50 doctors, 28 (56%) cited a lack of understanding the mechanism as a reason for not reporting ADRs. Moreover, 7 nurses and 7 pharmacists agreed with the doctors on this matter. 11(22%) doctors agreed that the reporting form is too complicated thereby acting as a barrier to ADR reporting. 2 (13%) nurses were in agreement also however no pharmacist agreed that the reporting form is too complicated. Interestingly, 11 (73%) of pharmacists agreed that no motivation was a factor that hindered ADR reporting. However, 36 (72%) doctors and 51 (64%) nurses did not agree that lack of motivation was a factor in reducing ADR reporting.

### Facilitators to enhance ADR reporting

**Fig. 2:**
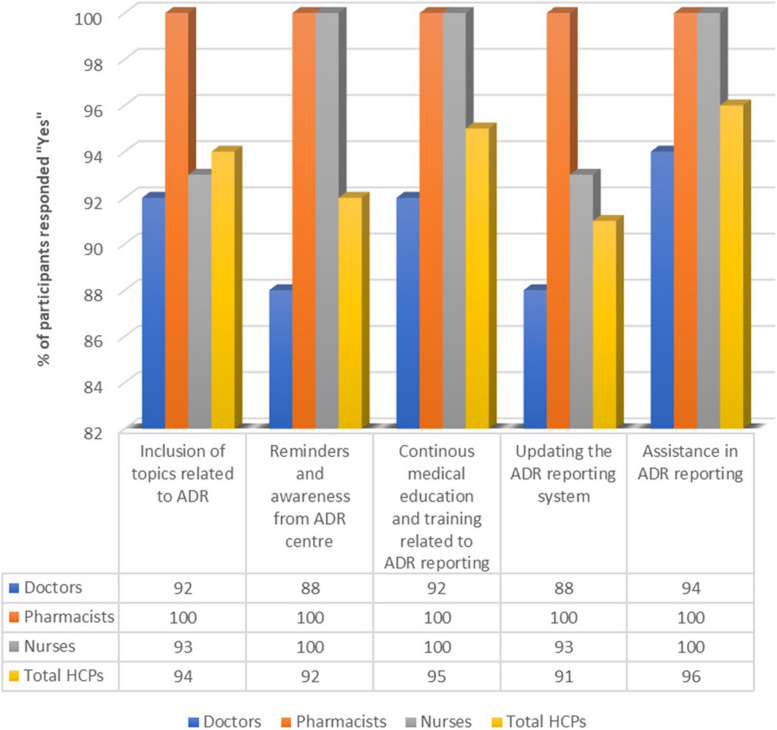
Facilitators on reporting ADRs

In our study, we outlined six strategies that are meant to encourage ADR reporting namely: “inclusion of topics related to ADR reporting in the curriculum”, “reminders and increased awareness from the ADR monitoring centre”, “continuous medical education/training related to ADR”, “updating the information on the ADR reporting system” and “assistance in reporting ADR” as shown in Fig. 2.

75 (94%) of all HCPs agreed to the strategy of inclusion of topics related to ADR reporting in the curriculum. Among these, 46 (92%) were doctors, 15 (100%) were pharmacists and 14 (93%) nurses concurred with this strategy. The prevalence of the HCPs who supported the reminders and increased awareness from the ADR monitoring centre was 74 (92%). 44 (88%) of the doctors agreed and all the nurses and pharmacists involved in this study agreed to this strategy. Similarly, all pharmacists and nurses involved in this study agreed to the strategy of continuous medical education, training related to ADR reporting. However, 4 (8%) of the doctors were in opposition towards this strategy.

With regards to updating the information on the ADR reporting system, 44 (88%) of the doctors and pharmacists agreed to this strategy. However, only 14 (93%) nurses agreed with this strategy. Furthermore, all nurses and pharmacists agreed to the strategy of using regulatory agencies to ensure a proper implementation of the ADR system. Only 40 (80%) of the doctors agreed to this strategy. A total of 77 (96%) of the HCPs involved in our study agreed that assistance is needed in the reporting of ADRs. 47 (94%) of the doctors said yes and all pharmacists and nurses likewise agreed that ADR reporting needs assistance. Significant associations were noted in variables such as “ADR is time consuming”, “fear of legal liability” and “no motivation” which had *p* values of 0.030, 0.001 and 0.002 respectively when associated with profession. Further, age was seen to be of significance when associating it with two barriers i.e. “ADR is time consuming” (*p* = 0.001), “fear of legal liability” (*p* = 0.002). Table 2 shows the significance of ADR associated barriers and facilitators as seen in this study.

**Table 2:** Significance of ADR associated barriers and facilitators

**Variables**	*p-value*		
	Profession	Gender	Age
**Barriers of ADR reporting**			
ADR is time consuming	0.030*	0.608	0.001*
Fear of legal liability	0.001*	0.431	0.002*
Lack of understanding of the reporting mechanisms	0.838	0.287	0.255
ADR reporting form is too complicated	0.148	0.306	0.704
No motivation	0.002*	0.281	0.786
**Facilitators of ADR reporting**			
Inclusion of topics related to ADR reporting in the curriculum	0.564	0.272	0.849
Reminders and increased awareness from the ADR monitoring centre	0.162	0.595	0.451
Continuous medical education/training related to ADR	0.639	0.797	0.477
Updating the information on the ADR reporting system	0.361	0.400	0.650
Assistance in reporting ADR	0.417	0.713	0.483

^*^p < *0.05* is statistically significant. Data expressed as percentages or mean

## Discussion

This study has assessed the barriers and facilitators of reporting ADRs among HCPs in Tamil Nadu. By addressing these barriers and leveraging on the supportive factors, healthcare practitioners will be able to improve the reporting process thereby contributing to patient safety. Furthermore, we aimed to understand the need for proper ADR reporting. Most of the participants were middle aged adults (mean age 29.27 years) and they had an average knowledge on ADR reporting. However, another study conducted by Singh et al. in North India revealed that majority of the young HCPs (nurses and doctors) lacked proper understanding on ADR reporting. In their study, only 14.3% of the HCPs were aware of the ADR reporting system in India. A possible reason for the difference in our study outcomes based on age could be the fact that we included individuals 45 years and older and additionally, a significant number of HCPs were between the age 26–35 years [36].

In our study, a total of 42 (52%) HCPs stated that they lacked understanding of the reporting mechanism. This is similar to the outcomes from a study done by Hardeep et al. where only 59% of the doctors had awareness of the national ADR reporting system [37]. Likewise, a meta-analysis by Bhagavathula et al. which included 18 studies done in India revealed that a total of 55.6% of the HCPs were not aware of the ADR reporting system [38]. A study by Khan et al. revealed that the lack of proper perception of new drugs (77%) and fear legal liability (73%) were the major barriers of ADR reporting in their study [39].

Furthermore, the prevalence of HCPs who believed reporting ADR is time consuming was 31% and 69% did not agree that ADR reporting is time consuming. These results are similar to a study done by Khan et al. where 43.4% were of the opinion that ADR reporting was not time consuming as opposed to the 32.8% who agreed that it was time consuming [40]. Another study conducted among MBBS students in Bihar showed that 13.79% of the third-year students believed ADR reporting to be time consuming while only 10.82% of the fourth-year students agreed with this statement [41]. A similar study showed that having a tight professional related schedule accounted for 29.60% of the unreported ADRs [42].

In our study, we discovered that 18 (22%) of the HCPs had a fear of legal liability and it acted as a barrier to ADR reporting. Only 9 (18%) of our doctors agreed to this statement and this is lesser compared to a study by Nadew et al. where 51.9% of the doctors believed that legal liability hindered them from reporting ADRs [43]. A study by Husain et al. concurred with the fact that legal liability particularly in the current scenario of medical practice has become a significant hindrance in ADR reporting [26]. Additionally, 16% of the HCPs involved in our study reported that the ADR report form is too complicated thereby acting as a hindrance to ADR reporting. A cross-sectional study by AlShammai et al. in Saudi Arabia showed that 61% of their respondents agreed to the suggestion that the ADR from is too complex to fill [44]. Only 11(22%) of the doctors agreed to this statement which is similar to the 32 (28.6%) of doctors who agreed that the ADR from is too complex to fill in a study by [45].

In this study, 75 (94%) of all HCPs agreed to the strategy of inclusion of topics related to ADR reporting in the curriculum. A study by Hadi et al. elaborated on the need for continuous educational development programs to strengthen ADR reporting [46]. Additionally, skill enhancement programs should be done among HCPs to enhance ADR reporting [47]. The prevalence of the HCPs who supported the reminders and increased awareness from the ADR monitoring centre was 74 (92%). A study by Kalikar et al. revealed the need for creating more awareness i.e. by using the clinical pharmacology subject among medical students in order to improve ADR reporting. This is after they did an educational intervention among 2nd year medical students and an improved knowledge, attitudes and practice were noted postintervention [48]. Moreover, a total of 77 (96%) of the HCPs involved in our study agreed that assistance is needed in the reporting of ADRs. This can be achieved through engaging skill enhancing programs and encouraging the physicians to report any suspected ADRs without looking down on any reaction.

### Limitations of the study

Conducting this study within a single healthcare institution is one of the limitations of our study. Moreover, the number of participants included in our study may not sufficiently give a clear representation of the barriers and facilitators of ADR reporting in India. Subsequent studies should be done in several centres and with more participants. Further, our study did not assess the influence of educational level of the responders.

## Conclusion

In summary, ADR identification and reporting is influenced by various factors encompassing both barriers and facilitators. Barriers such as time constraints, workload pressures and competing priorities often hinder HCPs from dedicating adequate attention to ADR reporting. Moreover, limited knowledge and awareness about the importance of pharmacovigilance and reporting systems can further impede the process. The inclusion of topics related to ADR reporting in the curriculum (i.e. clinical pharmacology) and increased awareness from the ADR monitoring centre were seen to be significant facilitators to enhance ADR reporting among health care practitioners.

## Supplementary Information

Supplementary Material 1.

## Data Availability

Data is provided within the manuscript or supplementary information files.
