# Supplementary material for: Barriers to and facilitators of healthcare professionals in ADR reporting in a tertiary care hospital in India
Source: BMC Health Serv Res. 2025 Jan 28;25:166. doi: 10.1186/s12913-024-12139-w (PMC11773872; doi:10.1186/s12913-024-12139-w)
Supplement: Supplementary file 1 — Supplementary Material 1. [file 12913_2024_12139_MOESM1_ESM.docx]

**
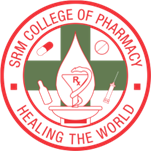

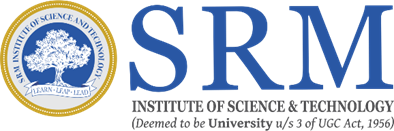
**

**Barriers and facilitators of healthcare professionals in Adverse Drug Reaction (ADR) Reporting**

**Data Collection Form**

**Date:**

**Name (Optional) ----------------------------------------------------------------------------------------------------**

**Age (years). -----------------------------------------------------------------------------------------------------------**

**Gender** ☐ Male ☐ Female

**Educational qualification -----------------------------------------------------------------------------------------------**

**Designation ----------------------------------------------------------------------------------------------------------------**

**Department ---------------------------------------------------------------------------------------------------------------**

**Work experience (Years)-------------------------------------------------------------------------------------------------**

**Profession**

☐ Doctor ☐ Pharmacist ☐ Nurse ☐ Others, please specify

**Are you aware of ADR reporting centre in our institute** ☐ Yes. ☐ No.

**Have you ever reported any ADR** ☐ Yes. ☐ No

**If yes how many times have you reported the ADR in the year 2023?**

**According to you what are the reasons for not reporting ADR**

**Reporting ADR is time consuming** ☐ Yes. ☐ No

**Fear of legal liability** ☐ Yes. ☐ No

**Lack of understanding of the reporting mechanism** ☐ Yes. ☐ No

**Reporting form is too complicated** ☐ Yes. ☐ No

**No motivation** ☐ Yes. ☐ No

**Please mention if any other reasons**

**What do you think can encourage healthcare professional to report the ADR**

**Inclusion of topics related to ADR in the curriculum** ☐ Yes. ☐ No

**Remainders and increased awareness from the ADR monitoring centre** ☐ Yes. ☐ No

**Continuous medical education and training related to ADR reporting** ☐ Yes. ☐ No

**Updating the information on the ADR monitoring system** ☐ Yes. ☐ No

**Regulatory agencies ensuring a proper implementation of the ADR system** ☐ Yes. ☐ No

**Assistance in ADR reporting** ☐ Yes. ☐ No

**Please mention any other additional thoughts**
